# Acquired aplastic anemia complicated with anti-glomerular basement membrane disease successfully treated with immunosuppressive therapy: a case report

**DOI:** 10.1186/s12882-022-02772-0

**Published:** 2022-04-07

**Authors:** Kenji Matsui, Wataru Kamata, Yasuhiro Mochida, Kunihiro Ishioka, Hidekazu Moriya, Sumi Hidaka, Takayasu Ohtake, Yotaro Tamai, Shuzo Kobayashi

**Affiliations:** 1 Kidney Disease and Transplant Center, Shonan Kamakura General Hospital, 1370-1, Okamoto, Kamakura, Kanawaga 247-8533 Japan; 2 Division of Hematology, Shonan Kamakura General Hospital, Kamakura, Kanawaga Japan

**Keywords:** Anti-glomerular basement membrane disease (anti-GBM disease), Aplastic anemia (AA), Human leukocyte antigen (HLA), Immunosuppressive therapy (IST), Case report

## Abstract

**Background:**

Aplastic anemia (AA) is a rare but fatal disorder characterized by pancytopenia due to bone marrow hypoplasia. Anti-glomerular basement membrane disease (anti-GBM disease) is an immune complex small-vessel vasculitis that presents as rapidly progressive glomerulonephritis and/or pulmonary hemorrhage. Although both involve autoreactive T cells that are partially triggered by human leukocyte antigen (HLA)-DR15, there have been no reports of their co-existence and the treatment strategy is not well understood.

**Case presentation:**

A 67-year-old woman presented with fever, malaise, and acute kidney injury with proteinuria and hematuria requiring hemodialysis. She was diagnosed with anti-GBM antibody disease based on high serum anti-GBM antibody titer and crescentic glomerulonephritis on a renal biopsy. Pulse administration of methylprednisolone (MP), oral prednisolone (PSL), and plasmapheresis were performed. Only 2 weeks after the diagnosis of anti-GBM disease, the patient developed pancytopenia requiring frequent blood transfusions. The blood cell count did not recover even 1 month after discontinuing the drugs that could cause pancytopenia. Bone marrow examination showed hypocellularity without abnormal infiltrates or fibrosis, which led to the diagnosis of severe acquired AA. Further HLA phenotyping revealed that she had HLA-DR15. Increased dose of PSL with the secondary MP pulse and the addition of cyclosporine improved pancytopenia. Although she remained dialysis-dependent, anti-GBM disease and pancytopenia did not recur for more than 2 years.

**Conclusions:**

We report the first case of acquired AA complicated with anti-GBM disease in an elderly woman with HLA-DR15, which was successfully treated with immunosuppressive therapy (IST). This report is valuable not only because it shows they may co-occur, but also because it provides a therapeutic option for this complex condition. It was also suggested that pancytopenia in patients with anti-GBM disease recalls serious hematologic diseases including AA that require immediate treatment based on bone marrow examination.

## Background

Aplastic anemia (AA) is a rare but fatal disorder characterized by pancytopenia due to bone marrow hypoplasia, with infection and bleeding being the main causes of death. Its 5-year survival rate is 38.1% in people aged ≥60 years [1]. Anti-glomerular basement membrane disease (anti-GBM disease) is an immune complex small-vessel vasculitis mediated by autoantibodies against GBM. It presents as rapidly progressive glomerulonephritis and/or pulmonary hemorrhage [2]. Both diseases share a common pathway involving dysfunction of T lymphocytes, although there have been no reports of their co-existence. Acquired AA occurs primarily due to indirect immune-mediated bone marrow destruction associated with activated autoreactive T lymphocytes and regulatory T-cell dysfunction [3]. One report demonstrated that approximately 10% of patients with AA had concomitant autoimmune diseases (AIDs), and the rate was > 25% for those > 50 years [4]. AA with systemic lupus erythematosus (SLE) [5], Sjogren’s syndrome [6], and antineutrophil cytoplasmic autoantibody (ANCA)-associated vasculitis [7] have been reported. On the other hand, anti-GBM disease involves not only B lymphocytes that produce specific antibodies, but also autoreactive T cells [8]. It has been reportedly complicated with hematological diseases involving lymphocyte abnormalities: T-cell large granular lymphocytic leukemia [9], Castleman disease [10], and hemophagocytic lymphohistiocytosis [11]. In addition, human leukocyte antigen (HLA)-DR15, an autoreactive T-cell trigger [12], has been implicated in the development of AA [13] and anti-GBM disease [14].

Here, we report the first case of acquired AA complicated with anti-GBM disease that responded well to immunosuppressive therapy (IST) including cyclosporine (CyA).

## Case presentation

**Fig. 1:**
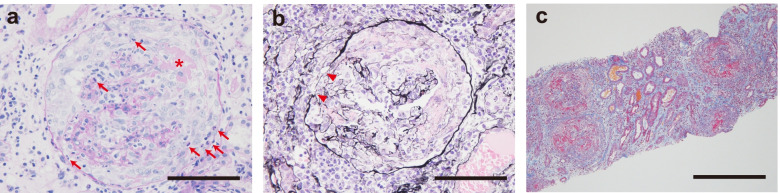
Light microscopic examination of the renal biopsy. **a**, **b** Glomeruli with circumferential cellular crescents with fibrinoid necrosis (asterisk in **a**), neutrophil infiltration (arrows in **a**), and ruptured Bowman’s capsules (arrowhead in **b**) and glomerular basement membranes (**a**, Periodic acid-Schiff stain; **b**, Periodic acid silver-methenamin stain). **c** Interstitial edema and mononuclear cell-predominant cellular infiltration (Masson’s Trichrome stain). Scale bars, 50 μm in **a** and **b**; 200 μm in **c**

A 67-year-old naturally healthy woman was admitted to the department of general medicine for a 2-week history of fever, malaise, sore throat, and elevated hepatobiliary enzymes. An antibiotic regimen was started for suspected acute cholangitis; however, her fever persisted and acute kidney injury occurred. On day 7, she was transferred to the department of nephrology on suspicion of vasculitis. She had no smoking history or was not taking daily maintenance medications. On examination, her body temperature was 38.5 °C and she had slight bilateral cost vertebral angle tenderness. She already had elevated serum creatinine (sCr, 1.56 mg/dL), proteinuria (2+), and microscopic hematuria (3+) on day 0. On day 7, sCr increased to 4.95 mg/dL. Serum anti-GBM antibody titer was 990 U/mL (negative < 3), whereas ANCA and antinuclear antibodies were negative. She was diagnosed with anti-GBM disease after a renal biopsy performed on day 8. It showed diffuse cellular crescents in glomeruli (65%, 22 out of 34) with fibrinoid necrosis, neutrophil infiltration, and ruptured Bowman’s capsules and GBMs (Fig. 1a and b), also with global sclerosis in 2 glomeruli (6%). There were interstitial edema and mononuclear cell-predominant cellular infiltration (Fig. 1c).

Hemodialysis was started, and thereby, she was treated with methylprednisolone (MP) pulse (1 g/day for 3 days) followed by oral prednisolone (PSL; initial dose, 60 mg/day) and plasmapheresis. Cyclophosphamide was not administered due to the absence of pulmonary hemorrhage and the low possibility of renal function recovery both clinically and pathologically [15].

**Fig. 2:**
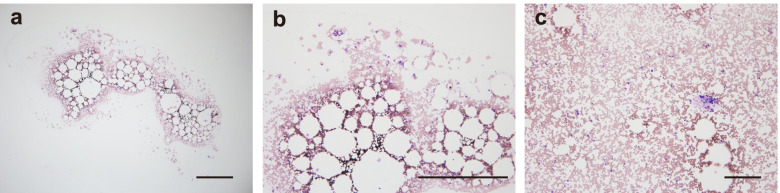
Hematoxylin-Eosin stain of the bone marrow. **a** A bone marrow biopsy shows low cellularity without abnormal infiltration or fibrosis. **b** The higher magnification of **a**. **c** A bone marrow aspiration smear does not contain atypical cells. Scale bars, 500 μm

On day 25, she developed pancytopenia that required frequent red blood cell and platelet transfusions. The respective minimum values were 4.0 × 10^3^/μL, 7.1/μL, 6.6 g/dL, and 9.0 × 10^3^/μL for platelets, neutrophils, hemoglobin, and reticulocyte, respectively. Initially, drug-induced pancytopenia was suspected, but pancytopenia persisted even 1 month after terminating trimethoprim/sulfamethoxazole and esomeprazole prescribed to prevent PSL side effects. Hemolysis, vitamin/iron deficiency, and viral infections were excluded. A bone marrow examination performed on day 62 was hypocellular with markedly decreased granulocytes, erythroblasts, and megakaryocytes. No abnormal infiltration or fibrosis was observed (Fig. 2a-c). Plasma thrombopoietin was 704 (> 320) pg/mL, ruling out myelodysplastic syndrome [16]. No peripheral erythrocytes of the paroxysmal nocturnal hemoglobinuria type or chromosomal abnormalities were identified. Therefore, the patient was diagnosed with severe acquired AA [17]. Further examination for HLA phenotypes revealed that she had A2/24, B48/52, Cw8/12, DR15/15, and DQ6/6.

**Fig. 3:**
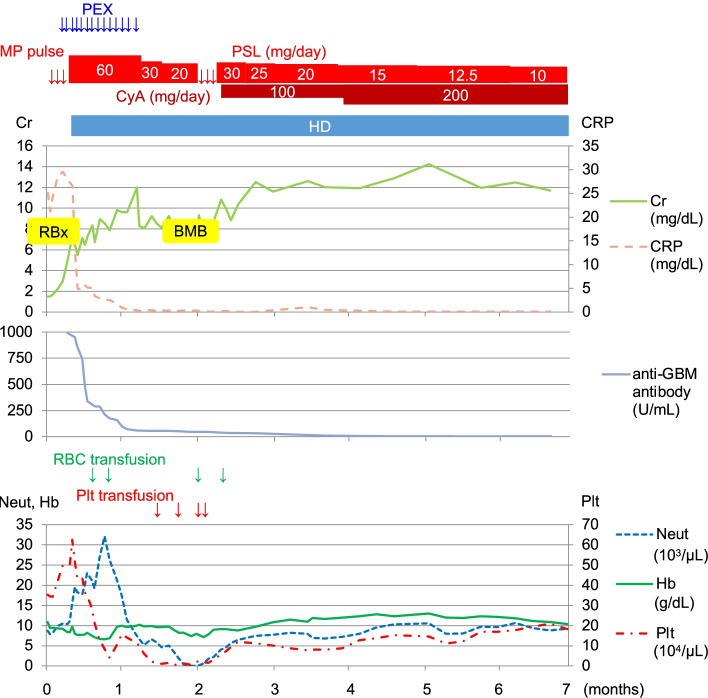
Presentation of the patient’s clinical course and treatment. The anti-GBM disease activity decreased with steroids and plasma exchange; however, the renal function did not improve. Two weeks after starting the treatment for anti-GBM disease, pancytopenia progressed. After the diagnosis of AA based on bone marrow examination, blood cells recovered with increased steroid dose and addition of CyA. BMB: bone marrow biopsy; Cr: creatinine; CRP: C-reactive protein; CyA: cyclosporin; GBM: glomerular basement membrane; Hb: hemoglobin; HD: hemodialysis; MP: methylprednisolone; Neut: neutrophil; PEX: plasma exchange; Plt: platelet; PSL: prednisolone; RBC: red blood cells; RBx: renal biopsy

After the secondary MP pulse, the PSL dose was increased from 20 to 30 mg/day. CyA was added keeping the trough level at 150–200 ng/mL [3]. CyA administration caused slight finger tremor, but the patient tolerated the treatment and was discharged home on day 80, after recovering from pancytopenia. Serum anti-GBM antibody titer became negative 16 months after the diagnosis of anti-GBM disease. After another 11 months, she received a living donor kidney transplant from her husband and has had no recurrence of anti-GBM disease or pancytopenia since then. (Fig. 3).

## Discussion and conclusions

In this report, we present the first case of acquired AA complicated with anti-GBM disease in an elderly woman with HLA-DR15, which was successfully treated with IST. Both AA and anti-GBM disease involve autoreactive T cells [3, 8], HLA-DR15 being a risk factor. The phenotype frequencies of DR15 in Japanese patients with AA and anti-GBM disease were 72% [13] and 94% [14], respectively, both significantly higher than those of the healthy controls. Reports from outside Japan had similar results [18–22]. However, HLA-DR15 alone insufficiently results in the development of these rare diseases [14]. The occurrence of anti-GBM disease in this patient without a smoking history may have been triggered by the preceding common colds [8]. Thereafter, pancytopenia occurred only 2 weeks after the anti-GBM disease diagnosis; it became worse after reducing PSL from 60 to 30 mg/day and was alleviated with IST enhancement including the addition of CyA, suggesting an association between AA and anti-GBM disease. The possibility that AA was induced by drugs, such as trimethoprim/sulfamethoxazole [23] or esomeprazole [24], cannot be excluded because drug-induced and idiopathic AA are not easily distinguished [25]. However, in this case, the blood cell count did not recover 1 month after discontinuing the suspected drugs, suggesting that these drugs were not the sole cause. As support, although the incidence of AA was relatively higher in sulfonamide users than in non-users, it was not statistically significant [26].

In this case, bone marrow examination was delayed because pancytopenia was initially thought to disappear with discontinuation of the suspected drugs. AA is a fatal disease that requires treatment based on immediate diagnosis; therefore, bone marrow examination should be immediately considered if pancytopenia appears in patients with anti-GBM disease.

For the treatment of acquired AA, IST consisting of anti-thymocyte globulin (ATG) and CyA is recommended; however, CyA alone can also be considered in the elderly patients [27]. HLA-DR15, especially genotype DRB1∗1501, is reportedly associated with good sensitivity to IST [28]. Nevertheless, the clinical course and treatment of AA complicated by AIDs are not well understood. One report found that the prognosis for AAs with or without AIDs was comparable [4]. Others have shown that AA with SLE [5] and Sjogren’s syndrome [6] responded to PSL or CyA without ATG. Combination therapy with ATG and CyA can remarkably cause strong immunosuppression, especially in patients with AID who have already received IST. A fatal brain abscess occurred in a patient with AA complicated by SLE who was treated with ATG, CyA, and high-dose PSL [29]. In our case, ATG was not administered because the patient was already in strong immunosuppression due to steroids and end-stage renal disease. AA was immediately responsive to CyA and increased steroids. Anti-GBM antibody titer was also controlled, although she had already reached renal death. Further reports are needed to establish a viable treatment strategy for AA with anti-GBM disease or other AIDs.

There are some limitations to this report: lack of HLA genotype identification and detailed analysis of lymphocyte populations before and after treatment, which would have been helpful to understand the pathogenesis of this case.

In conclusion, we report the first case of acquired AA complicated with anti-GBM disease, successfully treated with IST. This report provides a therapeutic option for this complex condition and suggests the necessity of immediate bone marrow examination against pancytopenia in anti-GBM disease patients.

## Data Availability

Not applicable.

## Abbreviations

AA: Aplastic anemia
AID: Autoimmune disease
ANCA: Antineutrophil cytoplasmic autoantibody
Anti-GBM disease: Anti-glomerular basement membrane disease
ATG: Anti-thymocyte globulin
CyA: Cyclosporine
HLA: Human leukocyte antigen
IST: Immunosuppressive therapy
MP: Methylprednisolone
PSL: Prednisolone
SLE: Systemic lupus erythematosus
